# Long-Term Survival and Kidney Function in Pediatric Patients Following Liver Transplantation: A 15-Year Retrospective Cohort Study

**DOI:** 10.3390/children9101544

**Published:** 2022-10-12

**Authors:** Rin Son, Sung Yun Suh, Yoon Sook Cho, Sandy Jeong Rhie

**Affiliations:** 1 Graduate School of Converging Clinical & Public Health, Ewha Womans University, Seoul 03760, Korea; 2 Department of Pharmacy, Seoul National University Hospital, 101 Daehak-ro, Jongno-gu, Seoul 03080, Korea; 3 Graduate School of Pharmaceutical Sciences, Ewha Womans University, 52 Ewhayeodae-gil, Seodaemun-gu, Seoul 03760, Korea; 4 College of Pharmacy, Ewha Womans University, 52 Ewhayeodae-gil, Seodaemun-gu, Seoul 03760, Korea

**Keywords:** long-term survival, kidney function, pediatric patients, liver transplantation

## Abstract

Long-term preservation of kidney function after liver transplantation (LT) has not been well studied. We thus evaluated the rates of kidney function preservation and long-term survival after pediatric LT. We also investigated the risk factors associated with the progression of chronic kidney disease (CKD). We conducted a retrospective study of 184 pediatric patients who had undergone LT from 2003 to 2018 at a university hospital. We collected demographics, primary indications for LT, liver disease scores, renal function test results, immunosuppressive drug prescriptions, and diagnosis of post-LT complications. The 15-year survival rate was 90.8%. Furthermore, the rate of kidney function preservation at 14 years post-LT in patients at high risk of renal disease was 79.3%, and that in those with less risk of kidney diseases was 96.0%. Arterial hypertension was an independent risk factor associated with CKD progression. However, when arterial hypertension was excluded, the use of cyclosporine and liver disease with renal involvement were risk factors for CKD progression. We found that kidney function after pediatric LT was well preserved. We encourage the early detection of underlying kidney involvement, routine monitoring of renal function for high-risk patients, active control of hypertension, and appropriate immunosuppressive regimens for pediatric patients with LT.

## 1. Introduction

Congenital liver diseases, such as biliary atresia and metabolic liver disease, are some of the major indications for pediatric liver transplantation (pLT). pLT has become the treatment of choice for pediatric liver diseases that possess a high potential risk of complications, such as acute liver failure and progression of end-stage liver diseases. Over the last few decades, advancements in surgical techniques, effective immunosuppressive regimens, and multidisciplinary management and monitoring strategies have been recognized for their contributions to decreased long-term complications and prolonged survival in the pediatric population after pLT [1,2]. With the success rate of pLT increasing up to 78–87% [1,3], the long-term preservation of kidney function after transplantation has been highlighted, especially in the pediatric population. It has been reported that physical health-related quality of life issues are decreased in patients whose renal function is decreased [4]. Interestingly, pediatric patients rarely present with chronic comorbidities prior to pLT, but they often face unwanted outcomes due to pLT, which could affect them later in life [5]. Much research is needed to prevent graft rejection while also avoiding major organ dysfunction to reduce systemic morbidities.

Renal dysfunction is a major post-pLT complication, and its prevalence was reported to be about 17.6% in a cross-sectional study from the Studies in Pediatric Liver Transplantation (SPLIT) registry [6]. However, others have reported the prevalence of chronic kidney disease (CKD) to be 3–33% at 7.6 and 10-year follow-up after pLT [7,8]. Another study reported that the 10-year cumulative mortality rate due to CKD in the pediatric population increased up to 79%, which resulted in patients requiring renal replacement therapy, such as dialysis and kidney transplantation, for the rest of their lives [9]. The maintenance of kidney function after pLT has varied in each study due to different numbers of patients, follow-up periods after transplantation, age at transplantation, and criteria of renal dysfunction. Further, previously published data on the high incidence of CKD and the progression to end-stage kidney disease after pLT showed associations with older age at the time of pLT, liver re-transplantation, reduced kidney function at baseline, and chronic nephrotoxicity of calcineurin [10,11,12]. Based on the considerable advancements in current surgical practice and pLT management, clinically applicable, timely, and reliable outcomes should be reevaluated to further preserve kidney function during the growth period after pLT.

This study evaluated the kidney function preservation rate in patients who survived at least a year after pLT for up to 15 years of follow-up and the long-term survival of all pediatric patients who received pLT. Moreover, the risk factors of CKD progression associated with pLT were also assessed.

## 2. Materials and Methods

### 2.1. Study Design and Data Collection

This was a retrospective cohort study of patients aged ≤18 years who underwent pLT for the first time. Patient data were collected from electronic medical records (EMRs) at the Seoul National University Hospital in Seoul, South Korea, for 2003–2018. Patients who had a history of pLT at other hospitals and underwent pLT with other organs simultaneously were excluded.

Demographic data included age at the time of pLT surgery, sex, height, weight, donor type, primary diagnosis, and estimated glomerular filtration rate (eGFR) at the time of LT. A sex-and age-based standard deviation (SD) score with reported height and weight was assigned by the Lambda–Mu–Sigma (LMS) method [13,14]. Pediatric end-stage liver disease (PELD) score components such as total bilirubin, serum albumin, and prothrombin time/international normalized time (PT/INR) were also collected. For patients older than 12, the model for end-stage liver disease score was primarily used to stratify LT waiting lists. However, those aged >12 and ≤18 were charted with PELD score in the EMRs, we thus collected PELD components. Renal function was determined by a bedside Schwartz equation when patients were 18 years old or younger, while a CKD-epidemiology collaboration-creatinine (CKD-EPI-Cr) formula was used for patients over 18 years old due to its superiority over a modification of diet in renal disease study (MDRD) formula evaluating kidney function [15,16,17]. The selected value of k was 0.413, and the height of each subject was used to calculate eGFR [15]. Other baseline characteristics were collected based on the following definitions: Graft rejection was defined as a rejection activity index (RAI) of ≥4 in histopathological diagnosis of biopsy via Banff schema [18]. Post-transplant lymphoproliferative disease (PTLD) was defined as positive results of immunohistologic staining of tissue biopsy. For kidney function preservation rate analysis, those who were administered rituximab were included [19]. Cytomegalovirus (CMV) infection was defined as positive results of CMV antigenemia assay (i.e., positive/200,000 white blood cells). Due to a mismatch of CMV serostatus (CMV immunoglobulin G: Donor+/Recipient−), pediatric patients have a higher risk for CMV infection than adults. In the recent period of this study, our center adopted a pre-emptive strategy for CMV infection after pLT. Thus, for CKD progression risk assessment, those who showed positive results on CMV antigenemia assay and received anti-cytomegalovirus therapy were included [20]. Arterial hypertension was defined as blood pressure > 95th percentile for the sex-, age-, and height-based reference ranges or administration of at least one anti-hypertensive medication post-pLT, excepting the use of anti-hypertensive medication during surgery and propranolol for pulmonary hypertension [11,21,22,23]. Hyperuricemia was defined as when the serum uric acid level was over the age-based reference range with at least two doses of anti-uricosuric agents [24]. The patient characteristics that were continuous variables were described by medians with interquartile ranges (IQRs).

### 2.2. Immunosuppressive Agent

Tacrolimus was dosed based on the target trough levels in the range of 8–12 ng/mL in the first month after pLT, and the dose was reduced to maintain the range of 6–8 ng/mL for the next six months. It was titrated down to 5 ng/mL thereafter. For cyclosporine (CsA), the initial target trough level was decreased from 250 (200–300) ng/mL to 225 (200–250) ng/mL within the first three months. Then, it was titrated down to 100–200 ng/mL within a year of pLT. The target trough level of CsA was set as under 100 ng/mL thereafter. Steroids were usually administered for the first six months and discontinued thereafter.

### 2.3. Survival and Kidney Function Preservation Rates

Survival was evaluated by measuring the time from the pLT to death or the end of the study period, whichever was earlier. After survival analysis, the patients were divided into two groups to compare the survival rates between patients with lower risk of renal involvement (non-KD group) and patients who had renal involvement, such as Alagille syndrome, hepatic fibrosis, and primary hyperoxaluria (KD group). A log-rank test was used to compare survival rates between the KD and non-KD groups [10,11,22,25,26].

For the kidney function preservation analysis, pediatric patients were excluded if they died during the first year following LT, as such deaths were thought to be driven by peri-transplant complications, such as graft dysfunction and hepatic artery complications, rather than kidney disease per se. In addition, those whose height was unmeasured, whose CKD stages were 4 or 5 prior to pLT, or who had acute renal impairment were excluded to analyze long-term results [10].

For the kidney function preservation rate analysis, patients were followed from the time of pLT to the time of renal function decline or the end of the study period, whichever was earlier. Kidney function decline was defined as a sustained decrease of eGFR to below <60 mL/min/1.73 m^2^ for three months [10].

The survival rates and kidney function preservation rates after LT were determined using the Kaplan–Meier method.

### 2.4. Factors Associated with CKD Progression

Risk factors associated with progression of stage 3 CKD or higher were analyzed by the Cox proportional hazards model with an unadjusted hazards ratio (HR) in univariate analysis and an adjusted HR (aHR) in multivariable analysis, along with a 95% confidence interval (CI) and *p*-value. The variables with *p* < 0.05 on univariate analyses were entered into the multivariable analysis (Model 1). For the assessment of other independent risk factors associated with CKD stage 3 (or higher), variables with *p* < 0.05 on univariate analysis, except the occurrence of arterial hypertension, were separately analyzed in another multivariable analysis (Model 2). In the multivariable analyses, statistically significant variables were estimated by backward elimination to investigate the role of interactions among variables. All statistical analyses were performed using IBM SPSS software version 25.

### 2.5. Ethics Statement

This study was reviewed and approved by the Institutional Review Board of Seoul National University Hospital (IRB No. H 2002 061 1100), and informed consent was waived owing to the retrospective nature of this study. The data were retrospectively collected using EMRs, and all datasets were protected in anonymized form.

## 3. Results

### 3.1. Patients’ Characteristics

In total, 190 pediatric patients received pLT at the hospital from 2003 to 2018. Six were excluded because one had a previous pLT at another center, and five had both liver and other solid organ transplants, four of which had liver and kidney transplants. Thus, 184 pediatric patients were included to analyze the cumulative survival rate after pLT. Eighty-nine (48.4%) were male, with a median age at pLT of 2.7 years old (IQR, 0.8–10.2), and the median follow-up duration was 8.5 years (IQR, 5.1–12.1). The median height was 87.7 cm (IQR, 70.0–133.0), and 44 (23.9%) patients were below −2 SD in the height-for-age Z-score. The median weight was 12.5 kg (IQR, 8.0–28.2), and 38 (20.7%) pediatric patients were below −2 SD in weight-for-age Z-score. Living donor recipients accounted for 110 (59.8%) cases. The most common indication for LT was biliary atresia, which was seen in 85 (46.2%) of pediatric patients. Twenty-two point eight percent (n = 42) of patients had liver diseases with concomitant kidney disease. Pre-transplant eGFR was 125 mL/min/1.73 m^2^ (IQR, 92.5–160.0). Only eight (4.3%) patients had ever used CsA as an immunosuppressive agent. Fourteen (7.6%) patients received pLT more than once during the follow-up period (Table 1).

### 3.2. Survival Rate of Study Population

**Figure 1:**
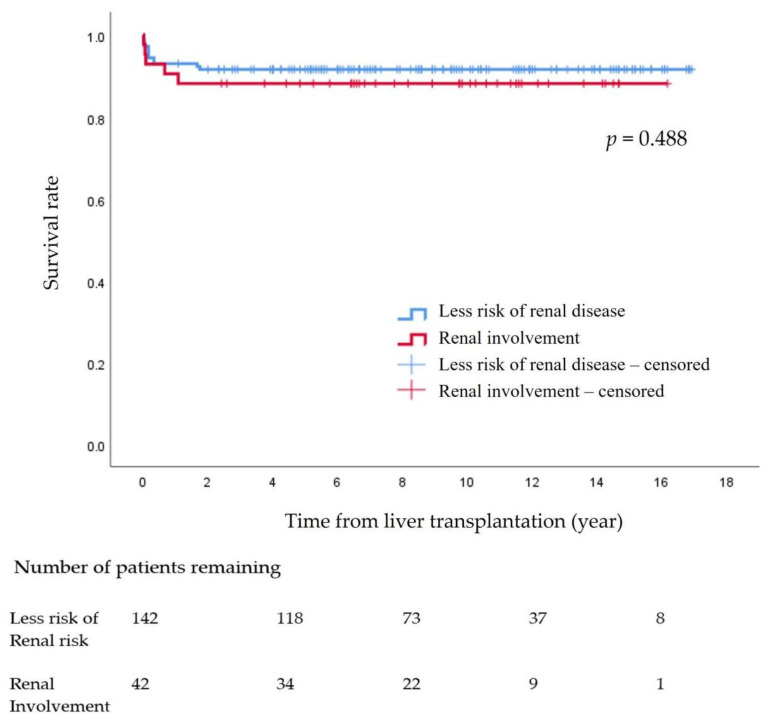
Survival rate after pediatric liver transplantation.

The survival rate at 15 years after pLT in the 184 pediatric patients was 90.8%. There were no significant differences between the non-KD group (91.5%) and the KD group (88.1%) (*p* = 0.488) (Figure 1)

### 3.3. Kidney Function Preservation Rate

After exclusions, 153 patients were included in the analysis. Patients were excluded if they died within a year post-pLT (n = 14), had no height records (n = 3), had stage 4 or 5 CKD before pLT and required renal replacement therapy at most at 2 weeks post-pLT (n = 7), or had acute renal impairment with fluctuation of renal function (n = 7). The median age at pLT was 2.3 years old (IQR, 0.8–10.4), and the median follow-up duration was 8.5 years (IQR,4.9–12.3). The median height and weight were 86.8 cm (IQR, 70.1–134.0) and 12.0 kg (IQR, 8.0–30.0), respectively. The median pre-transplant eGFR was 130.0 mL/min/1.73 m^2^ (IQR, 101.0–161.7). Biliary atresia was the most common indication for pLT, accounting for 52.3% (n = 80). Twenty-nine patients (19%) had liver disease with a potential risk of the following renal complications: Metabolic disease (glycogen storage disease (n = 9), tyrosinemia (n = 1), Wilson’s disease (n = 7)), cholestatic disease (Alagille disease (n = 8)), and congenital hepatic fibrosis (n = 4). Twenty patients were newly prescribed anti-hypertensive medications during the follow-up period. Ten patients (50%) were administered either calcium channel blockers, angiotensin-converting enzyme inhibitors, or beta blockers, and the rest (n = 10) had a combination regimen that included the above-mentioned drugs (Table 2).

**Figure 2:**
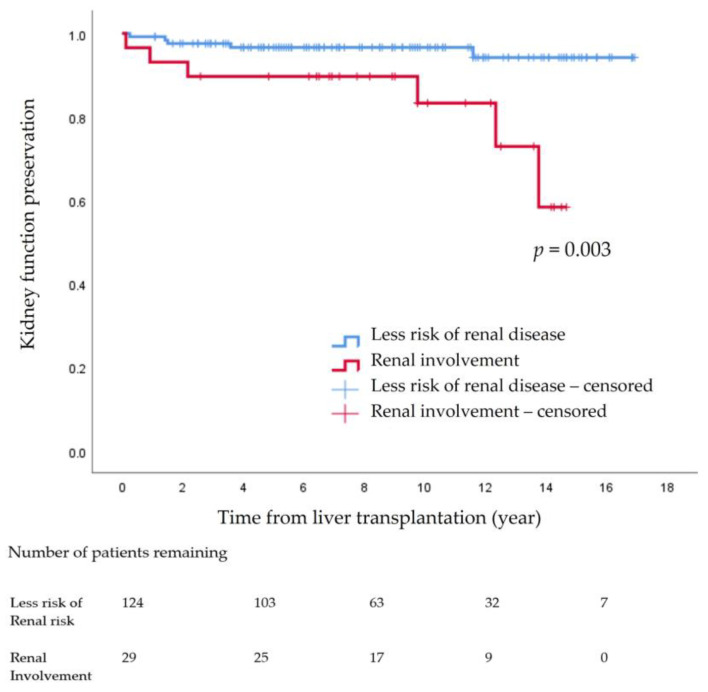
Kidney function preservation after pediatric liver transplantation.

The 14-year cumulative incidence rate of declining kidney function indicated that CKD ≥ stage 3 progression was 7.2% (n = 11). Among the 11 patients, one patient from the non-KD group (n = 5, 4.0%) underwent kidney transplantation, while in the KD group (n = 6, 20.7%), one patient underwent kidney transplantation, and the other received hemodialysis. The 14-year kidney function preservation rate was significantly different between the non-KD group (96.0%, n = 119) and the KD group (79.3%, n = 23) (*p* = 0.003) (Figure 2).

### 3.4. Factors Associated with CKD Progression

In the multivariable Model 1, new onset of arterial hypertension after pLT indicated a 14-fold increased risk of progression of stage 3 CKD or higher (aHR = 14.0, 95% CI, 4.1–48.3, *p* < 0.001). The use of CsA (aHR = 8.7, 95% CI, 2.4–31.8) and liver failure with concomitant renal involvement (aHR = 3.6, 95% CI, 1.0–12.4) were also significantly associated with the development of stage 3 CKD or higher (Table 3).

## 4. Discussion

In this retrospective long-term cohort study, we found that the survival rate after pLT was 90.8% for a 15-year follow-up period. According to the Korean Network for Organ Sharing (KONOS, a mandatory national data registry designed to obtain comprehensive information on the waiting and allocation process before transplantation) data from 2000–2015, the 15-year survival rate was 85.5% [27]. Considering that ours is a high-volume center with over 10 pLT cases per year, our study also confirmed that the survival rate after LT has improved. Given the improved success rate of pLT, long-term preservation of kidney function has attracted greater attention, especially in the pediatric population.

Our study showed that the post-transplant kidney function preservation rate, which was defined as no decrease of eGFR to below <60 mL/min/1.73 m^2^ for three months, was 96.0% in the pediatric patients who were less likely to have renal involvement prior to pLT. Sato et al., who had a similar endpoint as our study, found that eight-year renal preservation in the non-KD group was 92.4% [25]. The results are comparable, but it should be noted that our study’s results have added value in reporting the long-term post-pLT kidney function preservation, which was consistently high, with five more years in the observation period than the previous study. However, for the KD group, we found a 14-year kidney function preservation rate of 79.3%, whereas the eight-year kidney function preservation rate in KD patients was reported as 47.7% by Sato et al. [25]. There might be several reasons for the improved outcomes. One may be the tight post-transplantation management protocols that our institution has upheld since 2003. They consist of multidisciplinary team approaches with physicians, coordinators, clinical nutritionists, pharmacists, and social workers. The protocols include a pre-transplantation risk assessment to prevent potential hepatic and renal complications and post-transplantation follow-up and referral programs for long-term care [1,28,29,30]. In our center, which is one of the leading medical centers, after LT is performed, the physicians in charge order specific consultations on medication, diet management, and more for patients before they are discharged. In addition, our multidisciplinary team has a weekly meeting for outpatients to further follow-up and provide members with a real-time online information-sharing system. Many studies and guidelines mention multidisciplinary teamwork approaches as effective treatment management approaches for the prevention of recurrence and rejection and for monitoring children and adolescent patients to maintain kidney function and ensure long-term survival after LT [29,30].

We used eGFR values to assess kidney function rather than measured GFR (mGFR) when evaluating the prevalence of progression of CKD with stage 3 or higher, which was 7.2% during the 14-year follow-up in this study. In contrast, Campbell et al. reported that the prevalence of CKD progression with the primary endpoint measured by mGFR was <90 mL/min/1.73 m^2^, and the CKD progression rate was 17.6% at the mean of 5.2 years post-pLT in the multicenter and cross-sectional study of SPLIT [6]. It is noteworthy that our study complied with the criteria of CKD stages based on the recommendation by Kidney Disease Improving Global Outcomes and used eGFR as an indicator of the initiation of chronic renal function decline [31].

Age did not show statistical significance in the kidney function preservation rate post-pLT when it was adjusted with other variables. This could be because age at transplantation worked as an effect modifier, not a risk factor associated with CKD progression post-pLT. Almost half of the patients in the risk analysis were younger than 12 years old and demonstrated biliary atresia as their indication for pLT (n = 80, 52.3%). In addition, multivariable analysis revealed that liver re-transplantation was not an independent risk factor of CKD progression in this study. However, Ruebner et al. showed that liver re-transplantation resulted in a 2.7-fold increase in the risk of end-stage kidney disease while noting the outcomes of renal replacement therapy, such as dialysis or kidney transplantation [10]. Meanwhile, only 5.2% of patients in our cohort underwent liver re-transplantation. Unfortunately, our study was not able to confirm re-transplantation as a predictor of renal function preservation, likely due to the low incidence of re-transplantation in our study compared to that of about 15% in a previous study [10]. However, it should be considered that re-transplantation has been reported as a negative predictor of mortality [1,3]. Therefore, until further research is conducted, it is important to provide high-standard post-transplant care to maintain the remaining kidney function as much as possible before re-transplantation.

Harambat et al. verified that potential renal involvement prior to pLT was associated with the occurrence of chronic renal insufficiency in 69 pediatric patients after pLT in 2008 [11]. Our study confirmed these results with a larger population and longer observation period. Such findings suggest the importance of target management of patients with pre-existing renal conditions against deterioration to chronic renal complications. In addition, further investigation on liver disease that is concomitant with kidney disease could be one way to pre-emptively distinguish and manage high-risk patients, thus predicting renal function preservation.

Calcineurin inhibitors of CsA and tacrolimus (FK506) are known to be associated with dose- and efficacy-limiting adverse events, including nephrotoxicity, which may diminish the overall benefits for long-term graft survival. In our Model 2, the use of CsA was an associated risk with CKD progression. In their 10-year study, Harambat et al. reported a 25.4% of incidence of mGFR < 60 mL/min/1.73 m^2^, whereas 60.9% of enrolled patients used CsA within a month after LT [11]. The low incidence rate of 7.2% for CKD progression in the above-mentioned study might be influenced by the trend shift of immunosuppressive agents. In fact, switching to CsA is recommended when a hyperglycemic event, diabetes, biliary cirrhosis recurrence, or autoimmune hepatitis occurs after LT [32,33]. It is important to closely monitor the status of the graft as well as renal parameters when switching the immunosuppressive agent from tacrolimus to CsA. If CsA is used below the expected level of immunosuppression, to minimize its nephrotoxicity, graft rejection may occur, leading to long-term complications. Therefore, caution in the choice of immunosuppressive agents and a well-organized care plan for pediatric patients are essential to minimize immunosuppressant-induced nephrotoxicity and maintain long-term management to preserve kidney function and improve survival.

A multicenter prospective cohort study of Korean CKD children is currently in progress [34]. Of the 322 patients enrolled, 27% showed arterial hypertension at the beginning of the study. Twenty patients (13.1%) had newly started anti-hypertensive medications post-pLT. Many previous studies have revealed that hypertension is closely related to impaired kidney function [35,36]. Harambat et al. showed that arterial hypertension is associated with chronic renal insufficiency through a univariate analysis [11]. Moreover, Herzog et al. recommended a calcium channel blocker therapy to patients at risk of renal injury or with a low pre-transplant GFR [22]. Pediatric hypertension is assessed by sex-, age-, and height-based reference ranges, and timely physical examination is required to manage blood pressure for both renal function and chronic complications. Our cohorts show the need for close monitoring of patients with elevated blood pressure and intensive blood pressure control. To prevent the progression of asymptomatic hypertension, it is essential for the medical team to closely monitor the blood pressure in pediatric patients and to start anti-hypertensive medication in a timely manner.

This study has several limitations. First, it is a retrospective single-center study. Some patients in earlier cases had missing values for height and were thus excluded from the analysis. However, we collected the population data of all pediatric patients with pLT surgery at our institution. The study hospital has over 1700 hospital beds and operates a major LT center in Korea with a high number of cases of pediatric LT annually. Another limitation is that eGFR was calculated differently for different age groups due to the retrospective data collection. Even though the CKD-EPI-Cr method showed its superiority over the MDRD formula, one cross-sectional study revealed that the Schwartz equation is more reliable for estimating GFR in adults with mild to moderate kidney impairment up to 40 years old than the CKD-EPI-Cr formula [37]. The transition of pediatric patients to adult medical services with long-term post-LT care should thus not be overlooked. Additionally, we did not include other nephrotoxic agents such as vancomycin, aminoglycosides, or amphotericin B. Moreover, changes in donor availability, surgical techniques, and diagnostic methods were not considered. These would hardly affect the results because this was a single-center cohort study, but they may warrant assessment of the impact of surgical and diagnostic methods and concomitant nephrotoxic antimicrobial agents in future studies.

Survival was analyzed in pediatric patients for a 15-year post-pLT observation period. Increased interest in recent years on long-term outcomes such as physical health-related quality of life issues in the pediatric population has been emphasized by this study’s analysis of kidney function preservation and its association with CKD progression. Our results suggest that aggressive pre- and post-transplant care strategies should be implemented, such as the early detection of renal involvement, arterial hypertension, and optimal medication treatment.

## 5. Conclusions

The status of survival and kidney function preservation rates were evaluated after pLT. This study suggested the following strategies to improve pediatric patients’ kidney function preservation after pLT: Diligent monitoring and the prevention of arterial hypertension after LT, the proper use of CsA, and the management of concomitant renal involvement.

## Figures and Tables

**Table 1:** Baseline characteristics of study patients (n = 184).

Variable		Number of Patients n (%)
Sex		
	Male	89 (48.4)
	Female	95 (51.6)
Age at LT, yr ^a^		2.7 (0.8–10.2)
	<1	58 (31.5)
	1≤, <12	92 (50.0)
	12≤, ≤18	34 (18.5)
Height, cm ^b^		87.7 (70.0–133.0)
Weight, kg ^a^		12.5 (8.0–28.2)
Height-for-age, Z score ^c^		
	>−2 SD (normal)	135 (73.4)
	≤−2 SD (impaired)	44 (23.9)
Weight-for-age, Z score ^c^		
	>−2 SD (normal)	146 (79.3)
	≤−2 SD (impaired)	38 (20.7)
Donor type		
	Living	110 (59.8)
	Deceased	74 (40.2)
Primary indication for LT		
	Biliary atresia	85 (46.2)
	Metabolic disease ^d^	29 (15.8)
	Other cholestatic diseases	28 (15.2)
	Fulminant liver failure	20 (10.9)
	Cirrhosis	8 (4.3)
	Malignant tumor	7 (3.8)
	Others ^e^	7 (3.8)
Pre-existing renal involvement ^f^		42 (22.8)
Pre-transplant eGFR, mL/min/1.73 m^2 b,^		125 (92.5–160.0)
	≥90	138 (75.0)
	60≤, <90	24 (13.0)
	30≤, <60	6 (3.3)
	<30	11 (6.0)
PELD component		
	Total bilirubin at baseline, mg/dL ^a^	11 (2.6–18.9)
	Serum albumin at baseline, g/dL ^a^	3.3 (2.8–3.8)
	PT/INR at baseline ^a^	1.36 (1.15–2.01)
Follow-up, yr ^a^		8.5 (5.1–12.1)
Immunosuppressive agent		
	Cyclosporine A	8 (4.3)
	Others ^g^	176 (95.7)
Number of transplants		
	1	170 (92.4)
	2	13 (7.1)
	3	1 (0.5)

eGFR: Estimated glomerular filtration rate, LT: Liver transplantation, PELD: Pediatric end-stage liver disease, PT/INR: Protime/international normalized time, SD: Standard deviation. ^a^. median (interquartile range, IQR). ^b^. missing: n = 5, 2.7%. ^c^. Z-score: Sex- and age-based standard deviation score. ^d^. Hemochromatosis, glycogen storage diseases, primary hyperoxaluria, tyrosinemia, or Wilson’s disease. ^e^. Congenital hepatic fibrosis, autoimmune hepatitis, autosomal recessive polycystic kidney disease, or atypical hemolytic uremic syndrome. ^f^. Alagille syndrome, atypical hemolytic uremic syndrome, congenital hepatic fibrosis, glycogen storage diseases, primary hyperoxaluria, tyrosinemia, or Wilson’s disease. ^g^. Others include tacrolimus only, tacrolimus + steroid + mycophenolate, steroid only, and everolimus.

**Table 2:** Baseline characteristics of the patients for kidney function preservation analysis (n = 153).

Variable		Number of Patients n (%)
Sex		
	Male	72 (47.1)
	Female	81 (52.9)
Age at LT, yr ^a^		2.3 (0.8–10.4)
	<1	50 (32.7)
	1≤, <12	75 (49.0)
	12≤, ≤18	28 (18.3)
Height, cm ^a^		86.8 (70.1–134.0)
Weight, kg ^a^		12.0 (8.0–30.0)
Height-for-age, Z score ^b^		
	>−2 SD (normal)	116 (75.8)
	≤−2 SD (impaired)	37 (24.2)
Weight-for-age, Z score ^b^		
	>−2 SD (normal)	122 (79.7)
	≤−2 SD (impaired)	31 (20.3)
Donor type		
	Deceased	59 (38.6)
	Living	94 (61.4)
Primary indication of LT		
	Biliary atresia	80 (52.3)
	Other cholestatic diseases	24 (15.7)
	Fulminant liver failure	12 (7.8)
	Metabolic disease ^c^	19 (12.4)
	Cirrhosis	7 (4.6)
	Malignant tumor	6 (3.9)
	Others ^d^	5 (3.3)
Pre-existing renal involvement ^e^		29 (19.0)
Pre-transplant eGFR, mL/min/1.73 m^2^ ^a^		130.0 (101.0–161.7)
	90≤	130 (85.0)
	60≤, <90	23 (15.0)
PELD component		
	Total bilirubin at baseline (mg/dL) ^a^	11.1 (2.9–17.1)
	Serum albumin at baseline (g/dL) ^a^	3.3 (2.8–3.8)
	PT/INR at baseline^a^	1.35 (1.17–1.89)
Follow-up, yr ^a^		8.5 (4.9–12.3)
Number of transplants		
	1	145 (94.8)
	2	7 (4.6)
	3	1 (0.7)
Immunosupressive agent		
	Cyclosporine A	6 (3.9)
	Others ^f^	147 (96.1)
Acute cellular rejection		
	None	129 (84.3)
	1 or more	24 (15.7)
Late-onset acute cellular rejection		13 (8.5)
Anti-cytomegalovirus therapy		79 (51.6)
Arterial hypertension		20 (13.1)
Rituximab therapy after post-transplant lymphoproliferative disease		17 (11.1)
Hyperuricemia		10 (6.5)

eGFR: Estimated glomerular filtration rate, LT: Liver transplantation, PELD: Pediatric end-stage liver disease, PT/ INR: Protime/international normalized time, SD: Standard deviation. ^a^. median (interquartile range, IQR). ^b^. Z-score: Sex- and age-based standard deviation score. ^c^. Hemochromatosis, glycogen storage diseases, primary hyperoxaluria, tyrosinemia, or Wilson’s disease. ^d^. Congenital hepatic fibrosis, autoimmune hepatitis, autosomal recessive polycystic kidney disease, or atypical hemolytic uremic syndrome. ^e^. Alagille syndrome, congenital hepatic fibrosis, glycogen storage diseases, tyrosinemia, or Wilson’s disease. ^f^. Others include tacrolimus only, tacrolimus + steroid + mycophenolate, steroid only, and everolimus.

**Table 3:** Multivariable analysis of factors associated with stage 3 chronic kidney disease progression (n = 153).

Variable	Hazards Ratio (95% Confidence Interval)	*p*-Value	Model (1)		Model (2)	
			Adjusted Hazards Ratio	*p*-Value	Adjusted Hazards Ratio	*p*-Value
Age at LT (ref. <1 yr)						
1≤, <12	2.7 (0.3–24.0)	0.377				
12≤, ≤18	12.5 (1.5–103.8)	0.020				
Liver disease with renal involvement	5.1 (1.6–16.8)	0.007			3.6 (1.0–12.4)	0.043
Liver re-transplantation	4.9 (1.1–22.9)	0.043				
Use of cyclosporine	12.6 (3.6–43.6)	<0.001			8.7 (2.4–31.8)	0.001
Arterial hypertension (new onset)	14.0 (4.1–48.3)	<0.001	14.0 (4.1–48.3)	<0.001		
